# Fungal Gut Microbiota Dysbiosis and Its Role in Colorectal, Oral, and Pancreatic Carcinogenesis

**DOI:** 10.3390/cancers12051326

**Published:** 2020-05-22

**Authors:** Karolina Kaźmierczak-Siedlecka, Aleš Dvořák, Marcin Folwarski, Agnieszka Daca, Katarzyna Przewłócka, Wojciech Makarewicz

**Affiliations:** 1 Department of Surgical Oncology, Medical University of Gdansk, 80-214 Gdańsk, Poland; wojmakar@wp.pl; 2 Institute of Medical Biochemistry and Laboratory Diagnostics, Faculty General Hospital and 1st Faculty of Medicine, Charles University, 12108 Prague, Czech Republic; aleshdvorak@gmail.com; 3 Department of Clinical Nutrition and Dietetics, Medical University of Gdansk, 80-211 Gdańsk, Poland; marcinfol@gumed.edu.pl; 4 Department of Pathology and Experimental Rheumatology, Medical University of Gdansk, 80-211 Gdańsk, Poland; agnieszka.ela@gumed.edu.pl; 5 Department of Bioenergetics and Physiology of Exercise, Medical University of Gdansk, 80-210 Gdańsk, Poland; kaasiaa73@wp.pl

**Keywords:** mycobiota, colorectal cancer, oral cancer, pancreatic cancer, gut microbiota, *Saccharomyces boulardii*

## Abstract

The association between bacterial as well as viral gut microbiota imbalance and carcinogenesis has been intensively analysed in many studies; nevertheless, the role of fungal gut microbiota (mycobiota) in colorectal, oral, and pancreatic cancer development is relatively new and undiscovered field due to low abundance of intestinal fungi as well as lack of well-characterized reference genomes. Several specific fungi amounts are increased in colorectal cancer patients; moreover, it was observed that the disease stage is strongly related to the fungal microbiota profile; thus, it may be used as a potential diagnostic biomarker for adenomas. *Candida albicans*, which is the major microbe contributing to oral cancer development, may promote carcinogenesis via several mechanisms, mainly triggering inflammation. Early detection of pancreatic cancer provides the opportunity to improve survival rate, therefore, there is a need to conduct further studies regarding the role of fungal microbiota as a potential prognostic tool to diagnose this cancer at early stage. Additionally, growing attention towards the characterization of mycobiota may contribute to improve the efficiency of therapeutic methods used to alter the composition and activity of gut microbiota. The administration of *Saccharomyces boulardii* in oncology, mainly in immunocompromised and/or critically ill patients, is still controversial.

## 1. Introduction 

**Figure 1:**
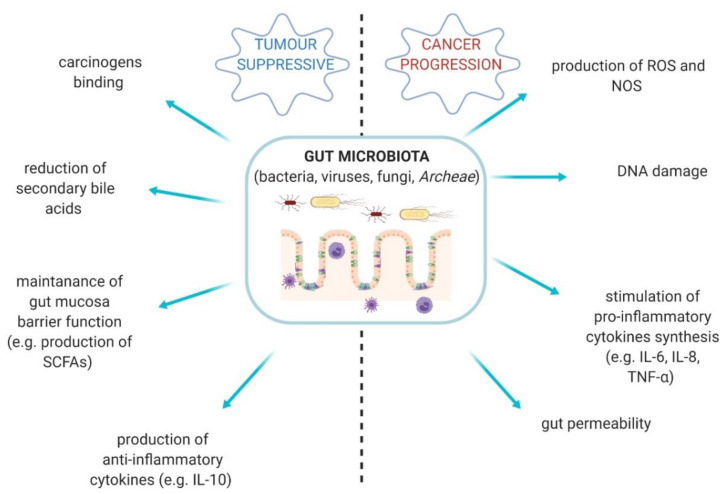
The diversity of gut microbiota activities—tumour suppressive conditions vs. cancer progression. SCFAs—short-chain fatty acids; ROS—reactive oxygen species; NOS—nitric oxygen synthase [2,3,4].

Gut microbiota is a complex ecosystem comprised of bacteria, fungi, viruses, and *Archeae* [1]. This vastly diverse community plays a pivotal role in human body [2]. Gut-resident microorganisms are able to produce an abundance of metabolites and bioproducts, which protect the homeostasis of the host and gut [3]. However, the quantitative and qualitative alterations in the composition of gut microbiota are described as gut dysbiosis and it takes part in the development of specific types of cancer. Some bacterial properties may, on the contrary, provide anti-tumour effects (Figure 1) [2,3,4].

The link between gut microbiota and gastrointestinal as well as oral cancers pathogenesis has been intensively studied with respect to bacteriome and virome changes [5]. Bacteria–virus interactions may prevent disease progression directly or indirectly via bacterial secreted products or stimuli, however, prolonged imbalance can cause chronic inflammation and cell transformation [6]. Extensive epidemiologic and pathologic studies have described the substantial impact of infectious agents on global cancer incidence [7]. Nevertheless, the association between fungal microbiota dysbiosis and specific carcinogenesis is still largely undiscovered, which is due to a relatively low abundance of intestinal fungi as well as lack of well-characterized reference genomes [8,9]. Unfortunately, the introduction of specific methodology is challenging [10] and consequently fungal microbiota is generally less explored than bacterial part. 

According to the American Cancer Society report from 2019, colorectal and pancreatic cancer were one of the most common cancers, both in men (1-lung, 2-prostate, 3-colorectal, 4-pancreatic cancer) and women (1-lung, 2-breast, 3-colorectal, 4-pancreatic cancer) [11]. In this paper, we focused only on gastrointestinal cancers and added oral cancers due to the fact that the oral cavity is the first part of digestive system. Therefore, this review is concentrated on the link between fungal microbiota dysbiosis and colorectal, oral, pancreatic carcinogenesis based on up-to-date studies. Additionally, we discussed the use of fungal strain probiotics in oncology. 

## 2. Gut Mycobiota in Healthy Gut 

Fungal microbiota is an integral part of gut microbial community; it is estimated that approximately 0.2% of microorganisms in human body are fungi [12]. The intestinal mycobiome encompasses both the resident community and its genome [12]. Its composition varies individually; however, *Candida*, *Saccharomyces*, and *Cladosporium* are the most common genera residing in the healthy human gut [13]. In the oral cavity, a low diversity of mycobiota is observed [14]. The most frequent fungi isolated from the healthy oral cavity are *Candida* spp. followed by *Cladosporium*, *Aureobasidium*, *Saccharomycetales*, *Aspergillus*, *Fusarium*, and *Cryptococcus* [15]. In Ghannoum et al.’s study, it was shown that *Candida* species were isolated from 75% of subjects [15]. *Candida albicans* is the most common opportunistic fungal pathogen isolated from human body. It causes superficial and chronic diseases. The infection of *C. albicans* often develops after antibiotic treatment [16]. 

The composition of fungal microbiota may be associated with age, gender, diet, and many others [1,17]. Strati et al. investigated the impact of age and gender on mycobiota [18]. In this study, faecal samples were taken from 111 Italian healthy volunteers (male *n* = 49, female *n* = 62, average age 10 ± 8.2 years). Fungi were detected in more than 80% of subjects and 349 different isolates were identified. The most common species (>10%) were *Candida albicans* (39.8%), *Rhodotorula mucilaginosa* (12.6%), and *Candida parapsilosis* (12.3%). The female subjects showed a higher number of fungal isolates (*p* < 0.005) and fungal species (*p* < 0.05) in comparison with male subjects. However, significant differences in the fungal population among the investigated age groups were not observed [18]. As Hoffmann et al. reported, the composition of fungal microbiota depends on diet, not only long-term diet, but also recent consumption [13]. Their research revealed a positive correlation between a high-carbohydrates diet and growth of *Methanobrevibacter* and *Candida*. In more detail, the abundance of *Candida* was strongly associated with recent consumption of carbohydrates; on the contrary, the abundance of *Methanobrevibacter* was associated to both long-term and recent consumption. However, negative correlation with the content of amino acids, proteins and fatty acids in the diet was observed in both species [13]. Overall, the gut mycobiota is more susceptible to changes in its composition in comparison to bacterial part of microbiota. On the other hand, disruption of the bacterial microbiota is a prerequisite for fungal overgrowth [19]. 

**Figure 2:**
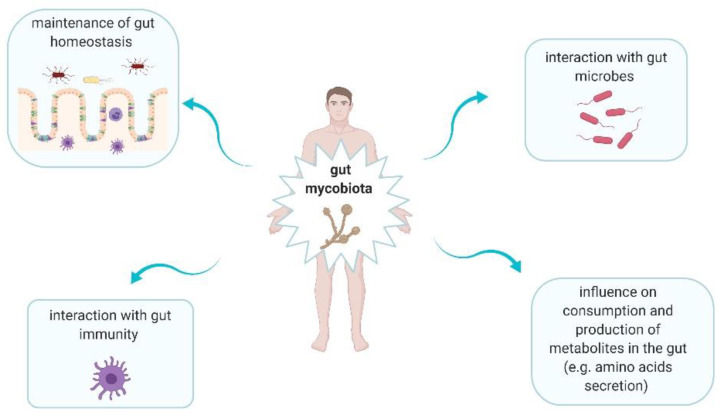
The main roles of gut mycobiota in human body [20,21].

Fungi are ubiquitous microbes that play significant roles in human gut (Figure 2) [20,21]. Intestinal fungi interact with bacteria. This interaction can be divided into three main categories, i.e., mutualism, commensalism, and competition [21]. Mutualism is observed when both microorganisms have an advantage from each other. This type of interaction between fungal and bacterial microorganisms rarely occurs [21]. Commensalism is defined as one microorganism gives an advantage to other but it does not receive this effect for itself [21]. For instance, it was shown that *C. albicans* enhanced the growth and proliferation of *Escherichia coli* K12 via supplying siderophore-like molecules to this bacteria [22]. Competition means that both microorganisms have a negative impact on each other. This type of interaction is observed between for instance *Pseudomonas aeruginosa* and *C. albicans*. *P. aeruginosa* inhibits *C. albicans* morphological transition to the hyphal form and kills *C. albicans* in this form. In turn, *C. albicans* secretes factors modulating *P. aeruginosa* virulence [23]. Fungi may alter consumption as well as production of metabolites in the gut, providing a positive or negative result. In Chiaro et al.’s study, it was assessed the impact of *Saccharomyces cerevisiae* colonisation on modulation of host purine metabolism exacerbating colitis in mice [24]. It was observed that *S. cerevisiae* enhanced host purine metabolism and consequently led to increase of uric acid level. Notably, this acid contributes to increased gut permeability [24]. Uric acid is a ligand of nod-like receptor family pyrin domain containing 3 (NLRP3) inflammasome, thus it promotes the production of interleukin (IL)-1β and IL-18 [24,25]. Moreover, fungi have an impact on immune development and homeostasis, due to their interaction with host immune cells [20]. Fungi can be recognized by five main receptors: Toll-like receptors (TLRs), C-type lectin receptors (CLRs), galectin 3 and NOD-like receptors (NLRs) on antigen-presenting cells (APCs), as well as NKp30 on natural killer (NK) cells [21]. The several pathways are triggered when fungi are recognized. It leads to production of mediators, such as interleukins (IL-1β, IL-6, IL-12, and IL-23), tumor necrosis factor-α (TNF-α), and interferon gamma (IFN-γ) [21]. Therefore, a strong association between intestinal immunity and mycobiota is observed [21]. 

## 3. The Link between Fungal Microbiota Dysbiosis and Carcinogenesis 

### 3.1. Colorectal Cancer

According to the World Health Organization, the number of deaths due to colorectal cancer (CRC) was approximately 862,000 in 2018 [26]. Currently, CRC is the third most common cancer worldwide with multifactorial etiopathogenesis, including genetic background as well as environmental factors, such as high-fat diet, low dietary fiber intake, consumption of red meat or sedentary lifestyle [27]. Gut microbes, which are influenced by diet, play a crucial role in the development of CRC [28]. It has been reported that several bacteria are involved in carcinogenesis of CRC, such as *Fusobacterium nucleatum*, *Bacteroides fragilis*, *Enterococcus faecalis*, *Streptococcus bovis*, *Escherichia coli*, *Helicobacter hepaticus*, and *Helicobacter pylori* [29,30,31]. These are known as colorectal cancer-associated pathogens. Additionally, the involvement of gut mycobiota in colorectal carcinogenesis has been increasingly recognized during last several years. 

A frequently studied substance in the context of nutrition are chitooligosaccharides (COS). Those oligomers are depolymerized from chitosan and have many biological activities, such as antimicrobial, antioxidant, anti-inflammatory, anti-tumour, and immunostimulatory [32,33]. Wu et al. have reported that COS modulates the intestinal bacterial microbiota and mycobiota, preventing the development of colitis-associated colorectal cancer [20]. In this study, it was noted that COS protected mice from CRC by reversing the imbalance of bacteria and fungi. It reduced the abundance of *Escherichia–Shigella*, *Enterococcus*, and *Turicibacter*; moreover, it increased the levels of *Akkermansia*, butyrate-producing bacteria and fungal genus—*Cladosporium* [26]. It should be emphasized that *Akkermansia municipla* improves the host metabolic functions as well as immune response and as Zhang et al. reported it may be a promising candidate as a probiotic [34]. In Nurhayati et al.’s study, it was also shown that COS has beneficial effects on probiotic bacteria as well as inhibitory effects on intestinal pathogens [35]. Moreover, Pan et al. reported that the concentration of SCFAs (short chain fatty acids) as well as the abundance of *Lactobacilli* and *Bifidobacteria* (commonly known as beneficial genera to the host) were significantly increased in the cecum of mice treated with COS [36]. Overall, the results of the above-mentioned studies confirmed that COS alters the bacterial as well as fungal microbiota and it may be useful in the prevention of CRC. 

Coker et al. characterised the enteric mycobiota in CRC [8]. In this study, faecal shotgun metagenomic sequences of 184 patients with CRC, 197 patients with adenoma, and 204 control subjects from Hong Kong were analysed. CRC-associated fungal markers and ecological changes were also validated in additional independent cohorts of 90 patients with CRC, 42 patients with adenoma, and 66 control subjects of published repository sequences from Germany and France. Faecal fungal dysbiosis associated with CRC was identified. The increase of *Basidiomycota*/*Ascomycota* ratio in patients with CRC in comparison with healthy individuals was noted. Moreover, *Malasseziomycetes* (fungal class) was enriched in CRC patients; however, *Saccharomycetes* (*Lypomyces starkeyi*, *Saccharomyces cerevisiae*) and *Pneumocystidomycetes* were depleted. It should be emphasized that *Saccharomyces cerevisiae* is the major component of the human gut microbiota and it plays a beneficial role in the gut. For instance, it has an anti-inflammatory properties due to induction of IL-10 production and tumor necrosis factor-alpha (TNFα) reduction [37]. Therefore, the decreased level of *Saccharomyces cerevisiae* in CRC patients is not desired. This study also demonstrated that abundance of 14 fungal biomarkers distinguished CRC patients from healthy subjects with validation in independent and ethnically different cohorts. These results suggest that fungal biomarkers might help to diagnose CRC [8]. The mycobiota dysbiosis was also investigated in Gao et al. trial including CRC patients (*n* = 74), colon polyp patients (*n* = 29), and healthy controls (*n* = 28) using high-throughput sequencing technology [38]. The biodiversity and composition of the fungi as well as the impacts of anatomic position and tumour stage on the mycobiota were analysed. The fungal dysbiosis in colon polyps and CRC was present. Visible mainly as increased *Ascomycota*/*Basidiomycota* ratio (similarly to above mentioned Coker et al.’s study) as well as increased proportion of opportunistic fungi—*Trichosporon* and *Malassezia*. Authors concluded that those changes may promote the progression of CRC. Moreover, the lower diversity and significant mycobiota alterations in early-stage tumours were observed. This study has uncovered a distinct fungal dysbiosis and an alteration in the fungal network. It should be noted, that they can play an important role in colon polyp and CRC pathogenesis [38]. 

Interestingly, Luan et al. also investigated the fungal microbiota dysbiosis in the intestinal mucosa of patients with colorectal adenomas [39]. This study included 27 subjects (average age—56.3 years, male—63%). The fungal microbiota of biopsy samples from adenomas and adjacent tissues was characterized using an Illumina HiSeq 2000 platform combined with the fungal internal transcribed spacer (ITS) region ITS1 and ITS2 primer pair. The most dominant phyla in both adenomas and adjacent tissues from all subjects were *Ascomycota*, *Glomeromycota*, and *Blasidiomycota*. Sixty genera were identified and *Phoma* and *Candida* (opportunist pathogens) represented an average of 45% of fungal microbiota. Moreover, if taking into consideration operational taxonomic unit (OTU) level, the decreased diversity in adenomas was noted. Similar to what was mentioned above in Gao et al.’s study, the disease stage was closely related to changes in the fungal microbiota. Therefore, this study presented the fungal microbiota profile in subjects with adenomas as a potential diagnostic biomarkers linked to different stages of the disease [39]. 

### 3.2. Oral Cancers 

Poor eating habits and oral hygiene, tobacco smoking, heavy alcohol consumption are the selected factors contributing to oral cancers development [40]. It has been shown, that microbial infections (fungal, bacterial, and viral) play a crucial role in the development of oral cancer [40]. It is estimated that more than 90% of mouth neoplasms are identified as oral squamous cell carcinoma (OSCC) [41]. 

Acetaldehyde (ACH) is the first metabolite of ethanol metabolism [42]. Due to its production by f.i. yeasts and acetic acid bacteria, it is naturally present in some alcoholic beverages [43]. ACH may be associated with the development of oral cancers. In Alnuaimi et al.’s study, it was compared the ability of biofilm-forming as well as production of hydrolytic enzymes and ethanol-derived ACH of oral *Candida* isolated from patients with oral cancer and matched non-oral cancer subjects [42]. It was observed that high ethanol-derived ACH-producing *Candida* isolated from patients with oral cancer was more prevalent in comparison to those with non-oral cancer (*p* = 0.01). Additionally, *Candida* isolated from patients with oral cancer presented significantly higher biofilm mass and biofilm metabolic activity as well as hydrolytic enzymes activity compared to subjects with non-oral cancer. Overall, these results suggest that *Candida* promotes the development of oral cancers due to its ability to produce hydrolytic enzymes and metabolize alcohol to carcinogenic ACH [42]. 

Makinen et al. examined the prevalence of *Candida* species in the saliva of OSCC patients and its effects on the mortality rate [44]. This study included 100 patients with OSCC and 75 age-matched controls (with no current or previously treated oral cancer). *Candida* genus was detected in 74% of patients with oral cancer, while *C. albicans* was the most common species (84%). *C. dubliniensis* (8%), *C. tropicalis* (4%), *C. glabrata* (3%), *C. parapsilosis* (3%), *C. sake* (3%), *C. krusei* (1%), and *C. guilliermondii* (1%) were other identified species. Notwithstanding, statistically significant effects of yeasts on mortality rate were not observed [44]. Another study demonstrated differences in mycobiota and bacterial microbiota between tongue cancer patients (TT—tumour tissue) and their matched normal oral epithelium (NTT—non-tumour tissue) [45]. It was noted that the most abundant bacterium phyla in both groups was *Firmicutes*, however, it was significantly increased in TT compared to NTT (48% vs. 40%, respectively; *p* = 0.004). *Bacteroides*, *Fusobacteria and Streptococcus* were also significantly increased in TT group in comparison to NTT. In the TT group, the abundance of *Aspergillus* was negatively correlated with *Actinomyces*, *Prevotella*, and *Streptococcus* and positively with *Aggregatibacter*. Patients with high T-stage (tumour stage) disease had significantly lower mean differences between TT and NTT compared with patients with low T-stage disease (0.07 vs. 0.21, *p* = 0.04) [45]. To conclude, this study demonstrated the differences in bacterial and fungal microbiota between patients with oral tongue cancer and their matched normal oral epithelium. This study also confirmed the association of gut microbiota with tumour stage indicating that elimination of bacterial and fungal microbiota dysbiosis may prevent or slow disease progression. 

It has been reported that inflammation caused by pathogens is involved in carcinogenesis. The increase of pro-inflammatory cytokines due to microbial infection of oral mucosa causes the inflammation [40]. For instance, *C. albicans* may promote carcinogenesis via several mechanisms, mainly triggering inflammation and induction of Th17 response [46]. The IL-17, produced by subpopulation of Th17 cells and the Th17-dependent signaling pathway, promotes NF-κB and the activation of Wnt pathway leading to tumor formation by proinflammatory environment creation [29]. Moreover, the increased expression of pro-inflammatory cytokines (mainly IL-6 and IL-8) are significant in inflammation and consequently in tumorigenesis of malignant cells. For instance, IL-6 exerts anti-apoptotic effect on malignant cells. The increased expression of IL-6 and IL-8 by oral cancer cells may be due to proteins glycosylated with *n*- or O-linked mannosyl residues, β-glucans and chitins on *C. albicans* cell’s wall, as well as the expression of secreted aspartyl proteinases (SAPs) from yeast [40,47]. 

### 3.3. Pancreatic Cancer 

The most common type of pancreatic cancer (and one of the most lethal cancers worldwide) is pancreatic ductal adenocarcinoma (PDAC) [48]. The risk factors for PDAC are chronic pancreatitis, diabetes, high-fat diet, obesity as well as gut dysbiosis [49]. *Helicobacter pylori* infection and oral pathogens, such as *Porphyromonas gingivalis*, *Neisseira elongata*, *Streptococcus mitis* contribute to PDAC development. Moreover, hepatotropic viruses potentially may also be involved in PDAC carcinogenesis [50]. The results of Aykut et al.’s study have confirmed that fungal microbiota promotes pancreatic oncogenesis [51]. It was noted that fungi migrate from the gut lumen to the pancreas taking part in pathogenesis of PDAC. The mycobiota promotes pancreatic oncogenesis via activation of mannose-binding lectin (MBL), which is a pattern recognition receptor (PRR) of the innate immune system. It binds to glycans of the fungal wall to activate the complement cascade [39,40]. MBL is required for oncogenic progression, whereas deletion of MBL or C3 in the extratumoral compartment-or knockdown of C3aR in tumour cells are both protective against tumor growth [51,52]. 

It is estimated that over 75% of pancreatic cancer cases are diagnosed at stage III/IV, thus at an advanced phase [53]. Currently, the treatment results of PDAC are still poor, radical surgery remains the only curative option (surgical resection followed by adjuvant chemotherapy) [54]. Early detection of PDAC gives the opportunity to improve the survival rate as well as patients’ quality of life. However, no well-recognized screening tools or biomarkers at the population level are available [49]. Nevertheless, Mendez et al. have reported that microbial alterations may be used as predictive markers for early detection of pancreatic cancer [55]. The abundance of *Proteobacteria* and *Firmicutes* in early stages of PDAC development was noted. Moreover, polyamine and nucleotide biosynthetic pathways were examined in detail to evaluate their importance in tumor progression. Therefore, the microbial changes and release of metabolites that foster host tumorigenesis are strongly related to occurrence of PDAC at early stages. Gut microbiota may potentially be treated as non-invasive tool for early detection of PDAC [55]. Notwithstanding, effective early diagnosis remains difficult and more specific biomarkers of this cancer are necessary [56]. The studies regarding the association of fungal microbiota dysbiosis with PDAC development are very limited. Therefore, further trials should also take into consideration fungal profile as a prognostic tool for PDAC. 

The summary of mechanisms in which the main fungal genera are involved in carcinogenesis of cancers mentioned above is presented in Table 1. 

## 4. Fungal Probiotics in Oncology 

### 4.1. S. boulardii—Characteristics and Properties 

*S. boulardii* CNCM I-745 is classified as non-bacterial probiotic microorganism belonging to *Saccharomyces cerevisiae* species [61]. It is the first yeast that has been studied for use as a probiotic strain in human medicine [62]. *S. boulardii* CNCM I-745 has multiple favourable properties, such as stability over a wide range of pH (including acidic conditions), resistance to antibiotics (due to its fungal nature), and promoting anti-inflammatory effects (e.g., reduction of pro-inflammatory cytokines, such as IL-8, TNF-α) [61,63,64,65]. *S. boulardii* CNCM I-745 has diverse mechanisms of action, affecting enteropathogenic microorganisms (adhesion on bacteria and their elimination) and the intestinal mucosa (trophic effects, epithelial reconstruction effects, anti-inflammatory action) [62,66]. 

*S. boulardii* may be used as a supportive treatment of e.g., antibiotic-associated diarrhea, *Clostridium difficile* infection (CDI), *Helicobacter pylori* infection, irritable bowel syndrome, inflammatory bowel diseases, dyslipidemia, and small intestine bacterial overgrowth in multiple sclerosis patients [66]. It should be noted that, that the studies’ results concerning *S. boulardii* or *S. boulardii* CNCM I-745 are similar—the yeasts’ effects are not strain- but species-dependent [66]. 

### 4.2. S. boulardii in Oncohematological Patients 

Nowadays, there are no guidelines on the routine use of *S. boulardii* in oncohematological patients due to the concerns that this probiotic strain may lead to severe invasive infection [67]. This is based on some case reports of *S. boulardii* or *Saccharomyces cerevisiae* sepsis [64,68,69,70]. Oncohematological patients often suffer from severe immune deficiency, thus, oral administration of probiotic products containing leaving yeasts may pose a particularly high risk of infection [71]. Furthermore, oral mucositis, which occur in up to 90% of patients preparing for hematopoietic cell transplantation (HCT), is common in these patients and it may contribute to yeast translocation through the oral mucous membrane into the bloodstream [67,72]. Consequently, it may cause fungemia and invasive infections [67]. However, the results of the most recent Sulik-Tyszka et al. retrospective analysis indicated that despite the colonisation of many oncohematological patients with *Saccharomyces* spp., cases of fungal sepsis caused by this species were not noted [67]. Notwithstanding, the *S. boulardii*, which may be administered mainly due to CDI or antibiotic-associated diarrhea to these patients, is still controversial and should be considered carefully.

### 4.3. S. boulardii in Immunosuppressed/Critically Ill Patients 

*S. cerevisiae* (thus, also *S. boulardii*) can cause different forms of invasive infections, for instance, if it is given to treat antibiotic-associated diarrhea [73]. The most important clinical syndrome caused by *S. cerevisiae* is fungemia [64]. It may occur in immunocompromised and/or critically ill patients [73]. Among others, the cases of fungemia in cancer patients have been reported by Anaissie et al. [74] and Aucott et al. [75].

Similarly, Appel-da-Silva et al. presented the case of an immunocompromised 73-year-old patient on chemotherapy who developed *S. cerevisiae* var. *boulardii* fungemia in a central venous catheter during treatment of antibiotic-associated pseudomembranous colitis with the probiotic containing *S. cerevisiae* var. *boulardii* [76]. Fungemia has resolved after the interruption of *S. boulardii* (Floratil^®^, Merck) administration. Similarly, as it was stated above, the authors emphasized that the use of *S. boulardii* should be discussed, due to inconsistent evidence of benefit in patients with *Clostridium difficile*-diarrhea and the high risk of fungemia in critically ill subjects [76].

### 4.4. S. boulardii—Prevention of Cancer Development 

Ulcerative colitis (UC) is described as chronic inflammatory disease of the colon. Patients with UC are at increased risk of CRC development. The repeated cycles of epithelial cells injury and repair contribute to UC carcinogenesis. During this process, the overproduction of proinflammatory cytokines (IL-6 and TNF-α) is observed. These cytokines are involved in all stages of carcinogenesis and consequently promote cancer progression. Wang et al., in an animal model study (C57BL/6 mice), have reported that *S. boulardii* treatment (in dose 5 × 10^7^ CFU/d for 12 weeks) reduced AOM/DSS-induced (azoxymethane/dextran sulfate sodium) UC carcinogenesis by decreasing the level of IL-6 and TNF-α [77]. Additionally, the authors have suggested that *S. boulardii* supplementation promotes the development of a healthier gastrointestinal microbiota that consequently helps to reduce the UC carcinogenesis induced by AOM/DSS [76]. Similarly, Fortin et al. have noticed that *S. boulardii* may have a potential role in colon cancer prevention [78]. *S. boulardii* cell wall extracts (crude insoluble glucan in doses of 0.5 and 1.0 mg/kg/day and a crude mannoprotein extract—0.3 and 3.0 mg/kg/day) were administered in rats (male F344 treated with 1,2-dimethylhydrazine) by gavage for 12 weeks. This study indicates that crude cell wall extract obtained from *S. boulardii* could prevent colon cancer in vivo, due to the potential influence on QR (quinone reductase) and β-glucuronidase modulation [78]. It should be noted that, β-glucuronidase is a lysosomal exoglycosidase involved in the degradation of glycosaminoglycans of the cell membranes and extracellular matrix of normal and cancerous colon tissues [79]. In Waszkiewicz et al.’s study, the significantly increased activity of β-glucuronidase in the serum of CRC patients in comparison to healthy subjects was noted; it indicates that serum β-glucuronidase activity has diagnostic value and potentially may be used in the diagnosis of colon adenocarcinoma [79].

## 5. Conclusions 

Mycobiota is an integral part of gut microbiota, but is relatively poorly studied. Nevertheless, the association between fungal dysbiosis and carcinogenesis is observed. 

Several specific fungi are increased in CRC patients and the diseases stage is closely related to a fungal gut microbiota profile, which is a potential diagnostic biomarker for adenomas. The major gut microbe causing an inflammation and consequently contributing to oral cancer development is *C. albicans*. Data investigations into the role of fungal microbiota in pancreatic carcinogenesis are still very limited. Therefore, there is a need to design and conduct a further studies also regarding the use of fungal microbiota profile as a potential prognostic tool to diagnose pancreatic cancer at an early stage, thus allowing for better outcomes for these patients. Notably, biomarkers should be validated in a wide range of the population, thus these studies must be conducted with an appropriate sample size. Moreover, future investigations should assess the impact of pre- and probiotics gut microbiota manipulation on fungal gut microbiota profile after anti-cancer treatment in patients with intestinal microbial imbalance. Further studies should also be conducted on homogenetic populations because gut microbiota and part of the mycobiota is ethnicity dependent. 

Additionally, the growing attention towards the characterization of not only bacterial or viral but also fungal microbiota composition and activity may contribute to achieve better efficiency of therapeutic approaches modifying gut microbiota, such as fecal microbiota transplantation. It is the most innovative method used to modify gut microbiota for instance, in patients with graft-versus-host disease after HCT. However, its safety is still controversial due to moderate (abdominal discomfort) to severe (CDI, death) adverse events. 

*S. boulardii* as a probiotic may be used in supportive treatment of several diseases, however, its administration to cancer patients (particularly in case of immunocompromised and/or critically ill subjects) should be considered carefully, due to the high risk of fungemia and consequent contribution to mortality. 

It should be noted that the link between fungal microbiota and cancer development as well as treatment is relatively new, promising and opens up new diagnostic and preventive options. 

## Figures and Tables

**Table 1:** The role of main fungal genera in carcinogenesis process.

Fungal Genus	Mechanisms in Cancer Development	References
*Candida*	➢ production of carcinogenic byproducts ➢ triggering inflammation ➢ induction of Th17 response ➢ increase the proliferation and activation of MDSCs (mainly G-MDSCs) ➢ metabolizing alcohol to carcinogenic ACH	[42,46,57,58]
*Malassezia*	➢ activation of mast cells and stimulation realising of proinflammatory cytokines (e.g., IL-6) via modulation of MAPK pathway ➢ activation of MBL	[39,51,59]
*Trichosporon*	➢ increasing the level of IL-6, TNF-α, IFN-γ, and G-CSF	[38,60]

Th17—T helper type 17 cells; MAPK—mitogen-activated protein kinase; MDSCs—myeloid-derived suppressor cells; G-MDSCs—granulocytic subtype of MDSCs; ACH—acetaldehyde; IL-6—interleukin-6; MBL—mannose-binding lectin; TNF-α—tumor necrosis factor α; IFN-γ—interferon γ; G-CSF—granulocyte-colony-stimulating factors.
